# An anatomical study of the subarachnoid space surrounding the trigeminal ganglion in horses—in preparation for a controlled glycerol rhizotomy in equids

**DOI:** 10.3389/fvets.2024.1424890

**Published:** 2024-07-18

**Authors:** Richard Becker, Kati Haenssgen, Christina Precht, Oleksiy-Zakhar Khoma, Ruslan Hlushchuk, Christoph Koch, Sabine Kaessmeyer, Mathieu de Preux

**Affiliations:** ^1^Department of Clinical Veterinary Medicine, Vetsuisse-Faculty, Swiss Institute of Equine Medicine (ISME), University of Bern, Bern, Switzerland; ^2^Division of Veterinary Anatomy, Department of Clinical Research and Veterinary Public Health, Vetsuisse-Faculty, University of Bern, Bern, Switzerland; ^3^Division of Clinical Radiology, Vetsuisse-Faculty, University of Bern, Bern, Switzerland; ^4^Institute of Anatomy, University of Bern, Bern, Switzerland; ^5^Graduate School for Health Sciences, University of Bern, Bern, Switzerland

**Keywords:** trigeminal-mediated headshaking, trigeminal cave, Meckel’s cave, magnetic resonance tomography, contrast cisternography, microtomography, histology, rhizotomy

## Abstract

**Introduction:**

Equine trigeminal-mediated headshaking is a painful neuropathic disorder comparable to trigeminal neuralgia in humans. The selective destruction of pain fibers within the trigeminal ganglion, called rhizotomy, is the surgical treatment of choice for idiopathic trigeminal neuralgia refractory to medical treatment in humans. The human trigeminal ganglion is enclosed by a dural recess called the Meckel’s or trigeminal cave, in which the ganglion is surrounded by a cerebrospinal fluid (CSF)-filled subarachnoid space. During glycerol rhizotomy, glycerol is percutaneously injected in this CSF-filled space. Until now, information about the anatomy of the dural recess and the subarachnoid space surrounding the trigeminal ganglion is lacking in horses. The aim of this study was to explore if a CSF-filled subarachnoid space around the trigeminal ganglion exists in horses.

**Materials and methods:**

Six equine cadaver heads were investigated for CSF accumulation around the ganglion with a 3 Tesla MRI. After anatomical dissection to expose the trigeminal root, a polymer-based radiopaque contrast agent was injected through the porus trigeminus into the subarachnoid space (cisternography). The exact delineation and the volume of the contrast agent accumulation were determined on subsequent micro-computed tomographic scans and segmentation. Finally, the distribution of the contrast agent within the subarachnoid space was examined histologically in three specimens.

**Results:**

In all 12 specimens included in this study, the trigeminal ganglion was surrounded by a subarachnoid space forming a trigeminal cistern. The mean volume of the trigeminal cave in this study was 0.31 mL (±SD: 0.11 mL). Distribution of the contrast agent along the peripheral nerves (i.e., ophthalmic, maxillary and/or mandibular nerve) was observed in 7 out of 12 specimens.

**Discussion/conclusion:**

A subarachnoid space surrounding the trigeminal ganglion exists in the horse and could be targeted for glycerol rhizotomy in horses suffering from trigeminal-mediated headshaking. However, the clinical relevance of contrast agent distribution along the peripheral nerves remains to be assessed.

## 1 Introduction

Trigeminal-mediated headshaking in the horse is a painful disorder characterized by violent, usually vertical shakes, flicks, or jerks of the head that can intensify under certain conditions, typically during exercise (1, 2). The clinical signs are believed to result from neuropathic pain induced by a sensory dysfunction of the trigeminal nerve (3), which can be severe enough to lead to early athletic retirement or even euthanasia for welfare reasons (2, 4). Equine trigeminal-mediated headshaking as a neuropathic disorder is in many aspects similar to human trigeminal neuralgia (3). Accordingly, therapeutic approaches from human medicine have been proposed for the treatment of affected horses. In humans, the selective surgical destruction of pain fibers within the trigeminal ganglion, called rhizotomy, is the surgical treatment of choice for idiopathic trigeminal neuralgia refractory to medical treatment (5).

In humans, the trigeminal ganglion is enclosed by an outpouching of the pachymeninx (dura mater). Therefore, two dural layers surround the crescent-shaped trigeminal ganglion and the trigeminal plexus (a term commonly used in human literature to describe the triangular-shaped plexual distribution of the trigeminal nerve with anastomoses between the nerve fascicles) (6, 7). This dural outpouching is commonly referred to as Meckel’s cave or trigeminal cave (8–10). Within the trigeminal cave, the ganglion is enveloped by the leptomeninx (pia mater and arachnoidea encephali) and the subarachnoid space located in between, with its content, the cerebrospinal fluid, forming the trigeminal cistern (6). This trigeminal cistern is the structure targeted with percutaneous glycerol rhizotomy for the treatment of trigeminal neuralgia (11). In this procedure, glycerol is injected percutaneously into the trigeminal cistern using a needle inserted through the foramen ovale. This allows glycerol to accumulate directly around the trigeminal nerve within the trigeminal cave, which then leads to selective destruction of its pain fibers, causing immediate pain relief (12, 13). The accuracy of needle placement in the trigeminal cave is mainly assessed on fluoroscopic images. The adjunction of contrast cisternography to native fluoroscopy, where the trigeminal cistern is filled with a radiopaque contrast agent, can help determine the appropriate volume of glycerol to inject for each individual (13).

To the authors’ knowledge, a subarachnoid space within a dural recess surrounding the trigeminal nerve and ganglion, similar to the trigeminal cave in humans, has not yet been described in the horse. Therefore, it remains unclear whether a CSF-filled trigeminal cistern exists and could be used as a target structure for glycerol rhizotomy in the horse. Nevertheless, percutaneous glycerol rhizotomies of the trigeminal ganglion have been attempted in horses, in an experimental setting and in clinical cases of trigeminal-mediated headshaking (14, 15). These rhizotomy procedures were performed using computed tomography (CT)-guidance to inject the glycerol directly into the ganglionic tissue but gave rise to numerous complications, including bleeding and meningitis (14). Neither the report on the experimental work nor that of the clinical cases included contrast studies to determine the distribution pattern of the injected substances or detailed information about the targeted anatomical structures. Furthermore, the reports did not mention the possibility of targeting a CSF-filled trigeminal cistern, as it is described for the treatment of trigeminal neuralgia in humans (14, 15).

The retrospective assessment of archived 3 Tesla (3 T) magnetic resonance imaging (MRI) scans of equine heads from the clinical radiology department of the Vetsuisse Faculty of Bern consistently revealed fluid accumulations of similar signal intensity to CSF surrounding the trigeminal ganglion. Therefore, we hypothesized that a subarachnoid space filled with CSF, comparable to the trigeminal cave in humans, also exists in horses.

Thus, the primary aim of this study was to explore the anatomical structure in which this fluid accumulation surrounding the equine trigeminal ganglion is located. The specific objectives were:

1. To describe the anatomy of the trigeminal ganglion and surrounding fluid-filled space in equine cadaveric heads using 3 T MRI.
2. To measure the volume of the fluid-filled space using contrast cisternography and subsequent analysis of micro-computer tomographic datasets.
3. To histologically assess the distribution of the casting material used for contrast cisternography within the trigeminal cave and its meningeal layers.

## 2 Materials and methods

### 2.1 Cadaveric specimens

Cadaveric heads of 6 client-owned horses, without known history of trigeminal-mediated headshaking or central nervous disease and euthanized for reasons unrelated to the purpose of the study were collected. Cadavers were donated after owners had signed an informed consent form, permitting the use of tissues and images for research purposes. An age range of 4–17 years, commonly reported for horses affected by trigeminal-mediated headshaking (16), was used as inclusion criteria to avoid age-related artifacts.

### 2.2 Magnetic resonance imaging

Magnetic resonance imaging was performed within 1 h of euthanasia. The heads were disarticulated at the level of the atlantooccipital joint and placed in supine position in the MR gantry for image acquisition. MR images were acquired with a 3 T MRI unit (Magnetom Vida, Siemens Healthineers, Zürich, Switzerland) and included a T1 weighted 3D reconstructable magnetization-prepared gradient-echo (MP-RAGE)-sequence in sagittal plane with a voxel size of 0.55 × 0.55 × 0.9 mm^3^, a T2 weighted TSE-sequences in sagittal and transverse planes and a T2 weighted fluid-attenuated inversion recovery (FLAIR)-sequence in transverse plane.

### 2.3 Contrast cisternography

**Figure 1:**
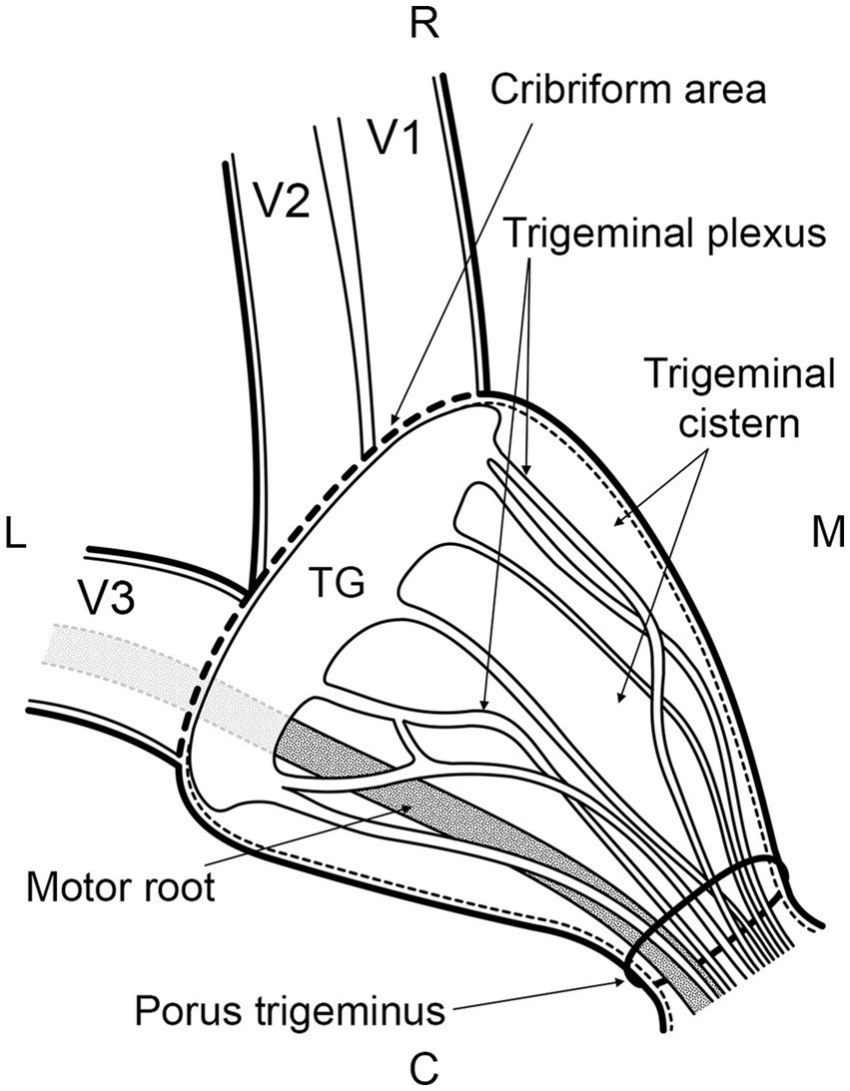
Schematic drawing in dorsal plane of a left trigeminal cave. The trigeminal nerve enters the trigeminal cave through the porus trigeminus whereafter it forms the trigeminal plexus. The trigeminal cistern is the cerebrospinal fluid-filled subarachnoid space within the trigeminal cave. The cerebrospinal fluid surrounds the fascicles of the trigeminal plexus. The nerve fascicles travel from the trigeminal ganglion (TG) through the cribriform area consisting of separate openings in the dura mater to unite and form the trigeminal branches—the ophthalmic nerve (V1), maxillary nerve (V2) and mandibular nerve (V3). In horses, V1 and V2 travel together as one nerve and separate before exiting the cranial cavity. The motor root runs alongside the sensory trigeminal root through the trigeminal cave but bypasses the trigeminal ganglion. Rostrally, it unites with the mandibular nerve. Bold line: dura mater; Thin dotted line: arachnoid; R: rostral; M: medial; C: caudal; L: lateral.

The first cadaver head was stored frozen after the MRI study for logistical reasons. All subsequent cadaver heads were not stored frozen and prepared for casting of the subarachnoid space immediately after MR imaging. Furthermore, casting was performed on the unfixed cadaver head to avoid formaldehyde-induced shrinkage artifacts. Following removal of the calvaria, the dura mater was carefully separated from the underlying arachnoid membrane to visualize its extent and to gain access to the dorsal aspect of the cerebrum. The latter was removed using a combination of digital blunt and sharp dissection to expose the pons and the trigeminal roots. The porus trigeminus (a term commonly used in human literature but not included in the Nomina Anatomica Veterinaria) was identified as the opening of the dural outpouching through which the trigeminal nerve enters the trigeminal cave at the lateroventral aspect of the cranial cavity, between the cerebrum and cerebellum (Figure 1). A slightly bent 25 G, 40 mm needle with a blunted tip was introduced under direct visual control between the arachnoid and the pia mater in the porus trigeminus to gain access to the subarachnoid space of the trigeminal cave. A polymer-based radiopaque contrast agent (μAngiofil^®^, Fumedica AG, Muri, AG, Switzerland) was then slowly injected into the subarachnoid space until backflow out of the porus was observed. During injection, the cadaver head was kept in a slightly tilted position, facing downwards rostrally, to promote rostral diffusion of the contrast agent. Contrast CT-cisternography was performed after a polymerization time of about 30 min. The cadaver heads were placed in a prone position in the gantry of a cone-beam CT (CBCT; O-arm 2, Medtronic, Louisville, Colorado), and a high-definition volumetric CBCT-scan using 120 kV and 150 mAs was acquired. Subsequently, the cadaver heads were fixed and stored in 4% formaldehyde. All the above-described procedures (i.e., MRI-scan, casting of the subarachnoid space, and CBCT-scan) were performed within 5 h and the cadaver heads were then submerged in a 4% formaldehyde solution.

### 2.4 Micro-computed tomography

After a minimum fixation time of 25 days in formaldehyde, the heads were cut with a bandsaw into blocks of 10 × 5 × 5 cm centered over each individual trigeminal ganglion. Each sample was subsequently scanned using a multiscale nanoCT system (SkyScan 2214, Bruker microCT, Kontich, Belgium). The x-ray source was set to a tube voltage of 110.0 kV and a tube current of 110 μA on average. The x-ray spectrum was filtered by an aluminum filter of 1 mm thickness prior to incidence onto the flat panel detector. For each sample, we recorded a set of 2 or 1 stacked scans overlapping the sample height, each stack was recorded with 3,601 projections of 3,072 × 1,944 pixels at every 0.1° over a 360° sample rotation. Every single projection was exposed on average for 760 ms. In total, 23 stacks were scanned. The acquired micro-computed tomographic (microCT) projection images were reconstructed by back-projection into a 3D stack of images with the NRecon software (NReconServer64bit, Bruker, MicroCT, Kontich, Belgium) using a ring artifact correction of 5 and a beam hardening correction of 80%. The whole process resulted in datasets with an isometric voxel side size of 30.0 μm. Using the CTVox software (Bruker, microCT, Kontich, Belgium), the virtual 3D-datasets were visualized. The volume of radiopaque casting agent injected in the subarachnoid space was determined by segmentation within the obtained 3D-datasets and using the CTAn software (Bruker, MicroCT, Kontich, Belgium). For each sample, segmentation and volume measurements were repeated three times by the same observer (R.B.) and each time in a different orientation on multiplanar reconstructions. The intraobserver variability of the measurements was assessed by performing Bland–Altman plots between the first and second, second and third, and first and third measurements. The collected data were analyzed using R statistical software (v4.1.2; R Core Team 2021). Bland–Altman plots were obtained via the ggplot2 R package (v3.3.3) (17).

### 2.5 Histology

Following microCT, 3 blocks from 3 different specimens were cut into slices of approximately 2 mm thickness for histological examination of the trigeminal ganglion and its surrounding meninges. In two blocks, the trigeminal ganglion was sectioned parallel to the porus trigeminus. In the last block, the slices were sectioned perpendicular to the porus trigeminus. The slices were subsequently fixed in 4% formaldehyde buffered solution, decalcified using Ossa Fixona^®^ (Diagonal GmBH & Co. KG, Münster, Germany), dehydrated with ethanol/Neoclear and embedded in paraffin. Slides of 2 μm thickness were further sectioned and stained with hematoxylin and eosin (HE). To facilitate differentiation of connective and meningeal tissue from nerve fascicles, slides were also stained with Masson-Goldner trichrome stain.

Cryofixation was performed in two slices originating from two different specimens. The slices were fixed in 4% formaldehyde buffered solution, decalcified using Ossa Fixona^®^ and then treated with 20% sucrose to avoid crystallization of water when freezing. Subsequently, they were loaded into a freezing holder, frozen at −80°C, further sectioned in slices of 5 μm thickness and stained with Masson-Goldner trichrome and HE. All sections were assessed and captured using a digital microscope (VHX 5100, Keyence, Osaka, Japan).

## 3 Results

### 3.1 Study population

The age of the 6 horses ranged from 11 to 16 years (mean 14 years, median 15 years). Included were 3 Arabian horses (2 mares, 1 gelding), 2 Warmbloods (1 mare, 1 gelding) and 1 Franches-Montagnes (mare). Body weight ranged from 415 to 607 kg (median bodyweight 537.5 kg).

### 3.2 Magnetic resonance imaging

**Figure 2:**
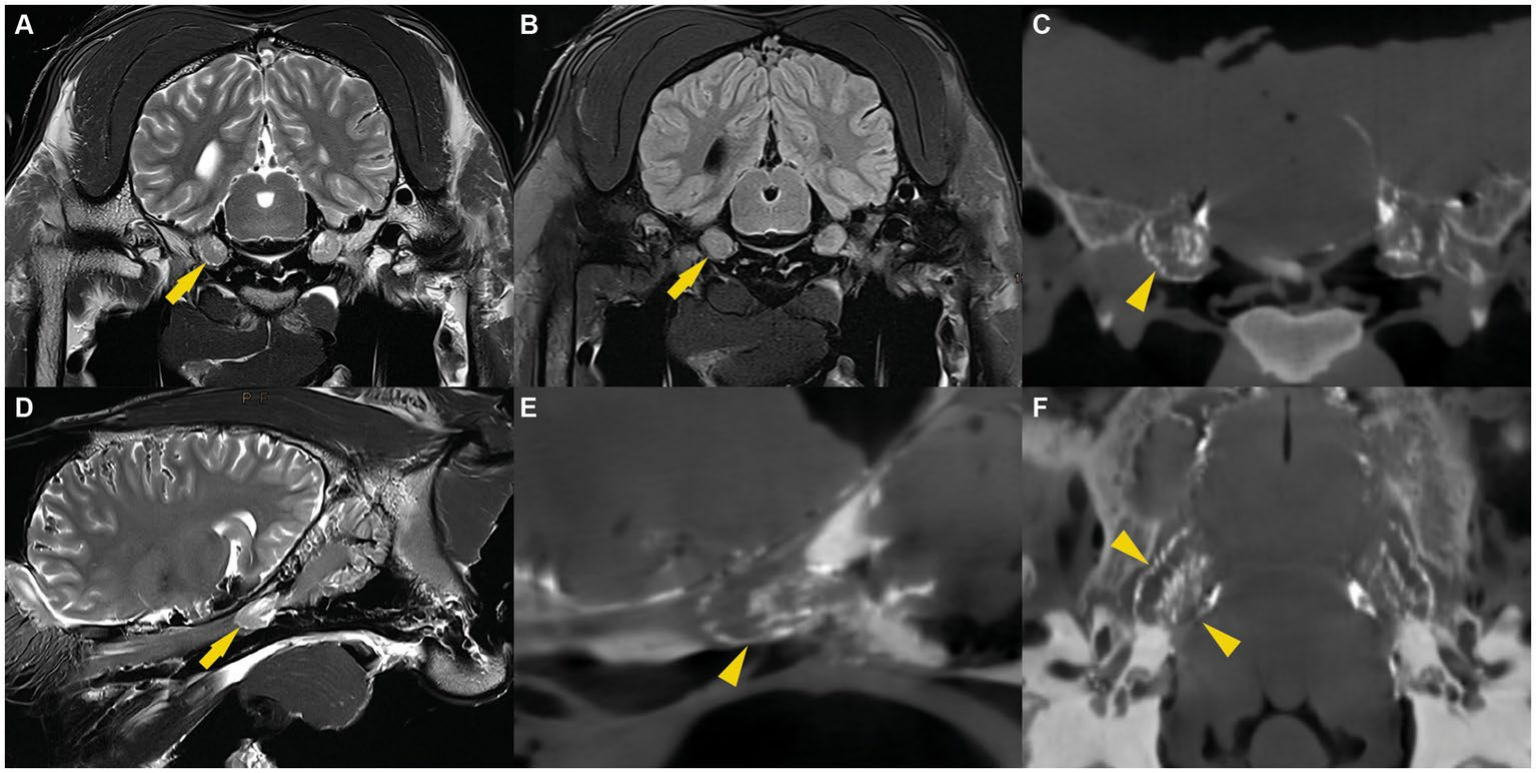
**(A)** T2w transverse, **(B)** T2w FLAIR transverse, **(D)** T2w parasagittal MR images and CBCT images reconstructed in transverse **(C)**, sagittal **(E)** and dorsal **(F)** plane illustrating the trigeminal ganglion surrounded by CSF (arrow) or casting material (arrowhead).

The MR images were assessed by a board-certified radiologist (C.P.) and consistently allowed bilateral visualization of fluid accumulation of similar signal intensity to CSF around the trigeminal ganglion. The CSF distribution pattern was similar in all specimens. On transverse cross-sectional T2 weighted TSE images, a subtle fluid demarcation line surrounded the trigeminal ganglion. In addition, a variable amount of fluid, arranged in a trabecular pattern, was identified in between nerve fiber bundles and the trigeminal ganglion. Both distinctive features were most pronounced on the axial aspect of the trigeminal ganglion. These images were compared with T2 weighted FLAIR sequences in transverse plane, which also showed a suppressed fluid signal surrounding the cerebrum, the cerebellum, and the trigeminal ganglion (Figure 2). This confirmed the CSF-nature of the fluid surrounding the trigeminal ganglion. On sagittal images, a hyperintense fluid signal in T2 TSE sequences and suppressed fluid signal in T2 weighted FLAIR sequences was detected all around the trigeminal ganglion. The fluid line surrounding the rostral aspect of the trigeminal ganglion consistently showed a tapered, convex shape. Caudally to the trigeminal ganglion but within the trigeminal cave, the fluid was arranged in a trabecular pattern resembling the distribution pattern on transverse images (Figure 2).

### 3.3 Contrast cisternography

On the CBCT-images, the radiopaque casting material was recognizable as a hyperintense, well-delineated line surrounding the trigeminal ganglion and located within the trigeminal cave. For each specimen, the (pre-cisternography) MR-images and the (post-cisternography) CBCT-images were compared. The distribution of the radiopaque casting material within the trigeminal cave was similar to the distribution of the CSF on the MR images, which suggested that the casting material had been injected into the (CSF-filled) subarachnoid space (Figure 2). In two trigeminal caves from two different cadaver heads, the shape of the radiopaque casting differed significantly from the other 10 trigeminal caves, including the contralateral ones. There, the casting material had undefined, irregular margins and did not extend as far rostrally as in the others. This presumably resulted from inadvertent perforation of the meninges during injection of the casting material.

The backflow of contrast agent during the injection through the trigeminal porus was visible in all specimens as a focal hyperattenuating signal on the CBCT-images. This hyperattenuating signal was located caudal to the trigeminal ganglion in the middle and posterior cranial fossa of the cranial cavity and caused no radiologic artifacts affecting the interpretation of the images.

### 3.4 Micro-computed tomography

**Figure 3:**
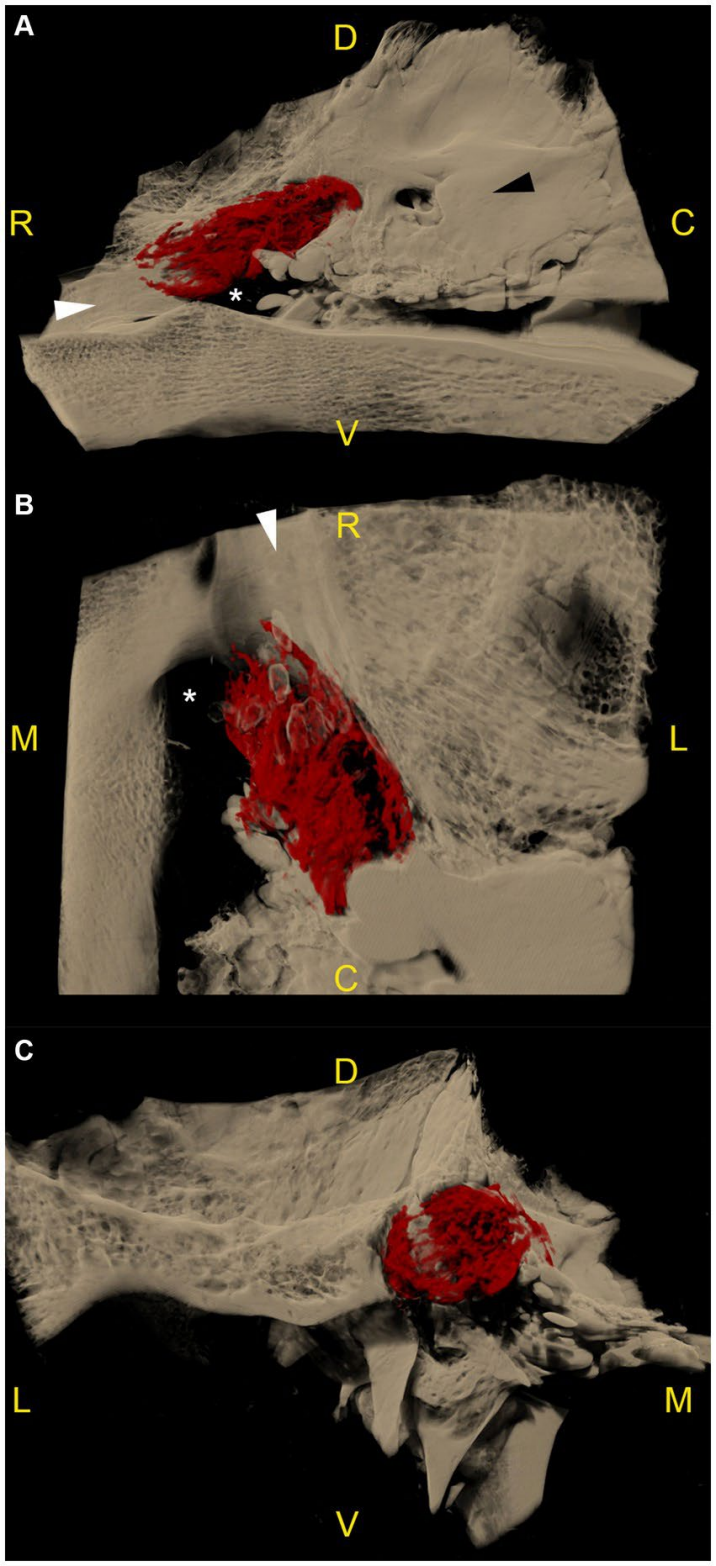
Microtomographic images of the contrast-filled subarachnoid space of a right equine cadaveric trigeminal cave without distribution of the contrast agent along the peripheral nerves. The contrast agent within the trigeminal cave is shown in red, and the surrounding bony structures are shown in light brown. **(A)** Trigeminal cave medial view, rostral is to the left. The accumulation of the contrast agent is less pronounced in the rostral third of the subarachnoid space and appears denser in its caudal two thirds. **(B)** Trigeminal cave dorsal view, rostral is to the top, medial is to the left. Note that the lateral aspect of the foramen lacerum (white asterisk) is covered by the trigeminal cave. **(C)** Transverse section through the trigeminal cave, rostral view, medial is to the right. The contrast agent mostly accumulates in the axial aspect of the subarachnoid space. The disruption of the contrast column in the lateral aspect of the trigeminal cave is due to the presence of the dense trigeminal ganglion. White asterisk: foramen lacerum; white arrowhead: alar canal; black arrowhead: petrous part of the temporal bone; R: rostral; D: dorsal; C: caudal; M: medial; L: lateral; V: ventral.

MicroCT-scans of both left and right trigeminal ganglia were performed on all 6 cadaver heads, resulting in 12 specimens available for segmentation. In all specimens, the accumulation of radiopaque casting material was located dorsally and laterally to the foramen lacerum, with its rostral border terminating over the rostral edge of the foramen lacerum, the lateral carotid incisure, the oval notch and the spinous notch, respectively. Laterally, the radiopaque contrast accumulation was closely associated to the internal acoustic meatus and the petrous part of the temporal bone of the middle cranial fossa (Figure 3).

The distribution pattern of the radiopaque casting material was similar in 10 out of 12 specimens (including the two frozen–thawed specimens), with a rostro-caudally oriented peanut-like/ovoid shape, and a notch halfway along its length caused by the branching of the mandibular nerve from the lateral surface of the ganglion. The contrast agent accumulated mostly in the caudal two thirds of the subarachnoid space. In the transverse section plane, contrast accumulation was mostly present on the axial aspect of the subarachnoid space (Supplementary Video S1). Several longitudinal filling defects were visible within the casting material, resulting in a trabecular pattern (Supplementary Videos S2, S3). In the rostral third, the contrast agent accumulated poorly around the rostral aspect of the trigeminal ganglion and the origin of the ophthalmic and maxillary nerve (Figure 3). The caudal boundary of the trigeminal cave varied in shape, due to the backflow of casting material through the porus trigeminus during the injection. However, the high resolution of the microCT scans allowed accurate identification of the oval-shaped porus trigeminus in all scans, and thus differentiation between backflow artifacts and the targeted subarachnoid space.

As already observed during CBCT-contrast cisternography, in 2 out of 12 specimens originating from two different cadaveric specimens, the shape of the radiopaque casting material accumulation varied considerably and was not comparable to that of the other 10 specimens, including the contralateral ones. In those specimens, the margins of the casting material were ill-defined, irregular, and did not reach as far rostrally as on the other specimens. These findings possibly resulted from inadvertent perforation of the meninges, which was already suspected during injection of the casting material.

**Figure 4:**
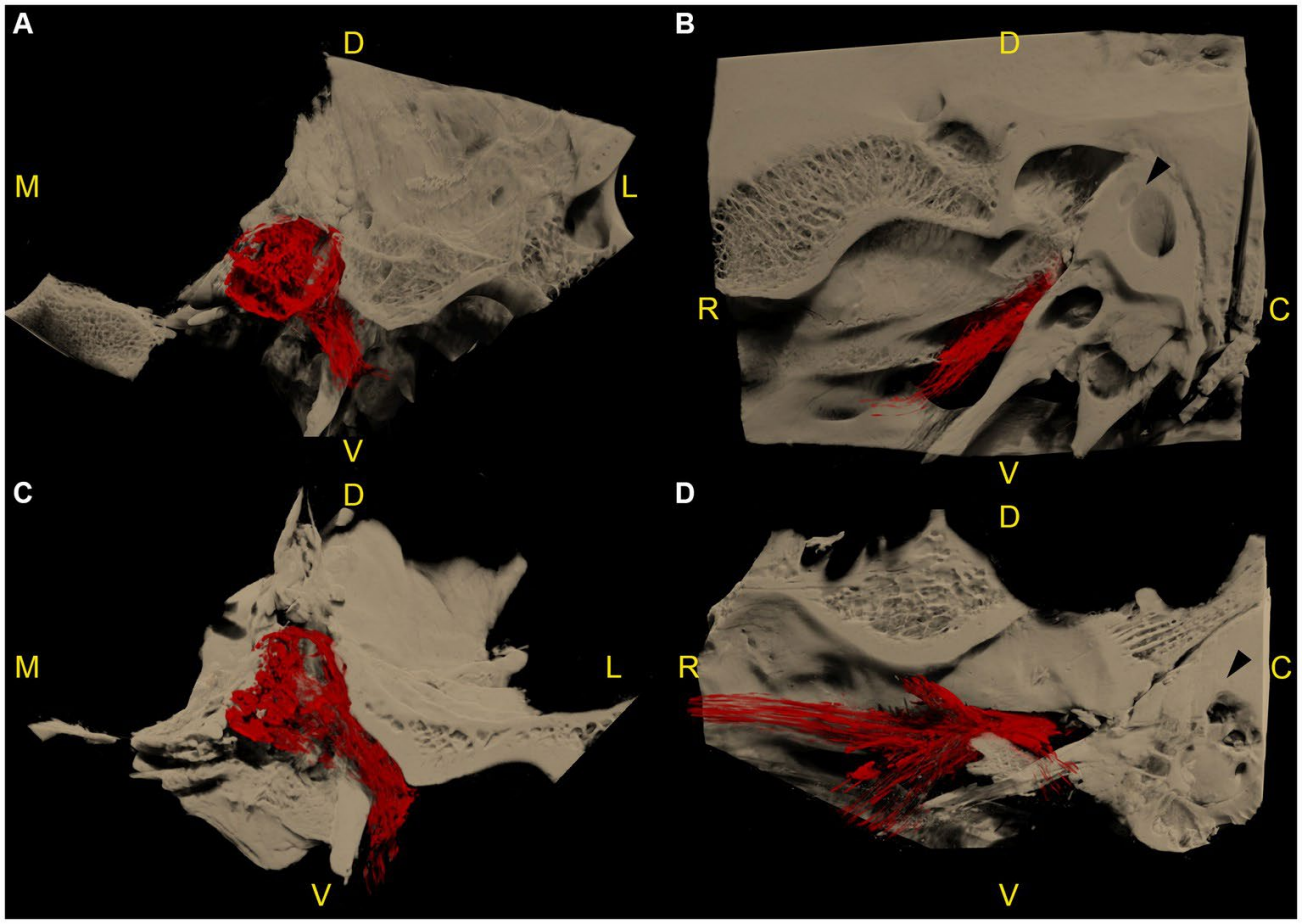
Microtomographic images of two left equine cadaveric trigeminal caves with distribution of the contrast agent along the mandibular nerve. The contrast agent within the trigeminal cave is shown in red, and the surrounding bony structures are shown in light brown. **(A)** Transverse section through the trigeminal cave of the first specimen, rostral view, medial is to the left. Note the distribution of contrast agent along the mandibular nerve through the foramen lacerum. **(B)** Same trigeminal cave as in **(A)**, lateral view, rostral is to the left. The contrast agent column surrounding the mandibular nerve fades briefly after it has passed through the oval incisure of the foramen lacerum. **(C)** Transverse section of the second specimen, same orientation as in **(A)**. The contrast agent column surrounding the mandibular nerve is longer than in **(A)**. **(D)** Same trigeminal cave as in **(C)**, same orientation as in **(B)**. The contrast agent column surrounding the mandibular nerve branches out in multiple directions after passing through the foramen lacerum. R: rostral; D: dorsal; C: caudal; M: medial; L: lateral; V: ventral.

**Figure 5:**
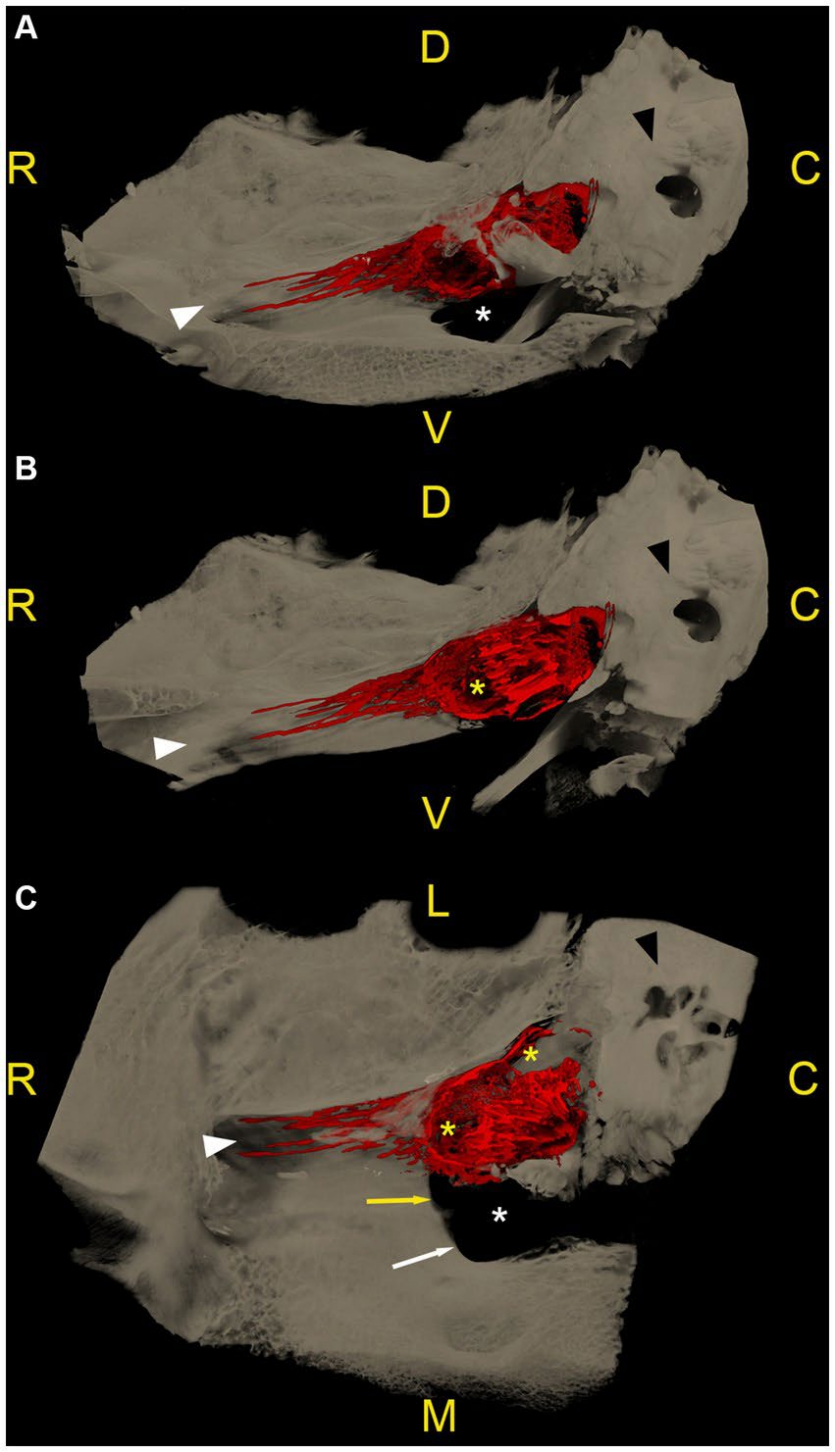
Microtomographic images of a right equine cadaveric trigeminal cave. The contrast agent within the trigeminal cave is shown in red, and the surrounding bony structures are shown in light brown. **(A)** Medial view, rostral is to the left. The porus trigeminus is clearly visible (right boundary of the contrast agent accumulation). There is a small amount of contrast agent that distributes along the lateral aspect of the maxillary nerve. The contrast agent column fades before the nerve exits the cranial cavity through the foramen rotundum. **(B)** Sagittal section of **(A)**. Note the focal loss of contrast agent (yellow asterisk) induced by the dense trigeminal ganglion. In the caudal aspect of the trigeminal cave the contrast agent accumulation shows a trabecular pattern because of the presence of fibers of the trigeminal plexus running through the trigeminal cistern. **(C)** Dorsal section of the trigeminal cave shown in **(A,B)**, rostral is to the left. Again, note the focal absence of contrast agent (yellow asterisk) induced by the trigeminal ganglion, and the trabecular structure of the subarachnoid space induced by the diverging fibers of the trigeminal plexus. White asterisk: foramen lacerum; white arrowhead: canalis nervi maxillaris; black arrowhead: petrous part of the temporal bone; white arrow: carotid notch; yellow arrow: oval notch; R: rostral; D: dorsal; C: caudal; M: medial; L: lateral; V: ventral.

In 7 out of 12 specimens, a variable amount of radiopaque contrast agent was distributed along the course of the proximal aspect of the main branches of the trigeminal nerve, in 3 out of 12 around the mandibular nerve (Figure 4), in 3 out of 12 around the maxillary and ophthalmic nerve (Figure 5), and in 1 out of 12 specimens around the three main branches of the trigeminal nerve. In one specimen, the contrast agent was not only distributed around the origin of the mandibular nerve, but also extended along the course of the mandibular nerve through the foramen lacerum and along the nerves branching off outside the cranial cavity (Figure 4; Supplementary Video S4).

Assessment of the Bland–Altman plots revealed a narrow limit of agreement, a consistent variability across the graphs and no systematic bias, thus highlighting the low intraobserver variability between the three measurements. Therefore, the mean value of the three measurements for each trigeminal ganglion was calculated and described as the mean volume of the radiopaque casting material.

The mean volume of the radiopaque casting material contained in each subarachnoid space ranged from 0.13 to 0.48 mL, with an overall mean volume (mean of the 12 mean values) of 0.31 mL (±SD: 0.11 mL). The two specimens exhibiting poor contrast agent distribution had the overall lowest mean values (0.13 and 0.16 mL). When excluding those two specimens, the overall mean volume reached 0.34 mL (±SD: 0.09 mL).

### 3.5 Histology

**Figure 6:**
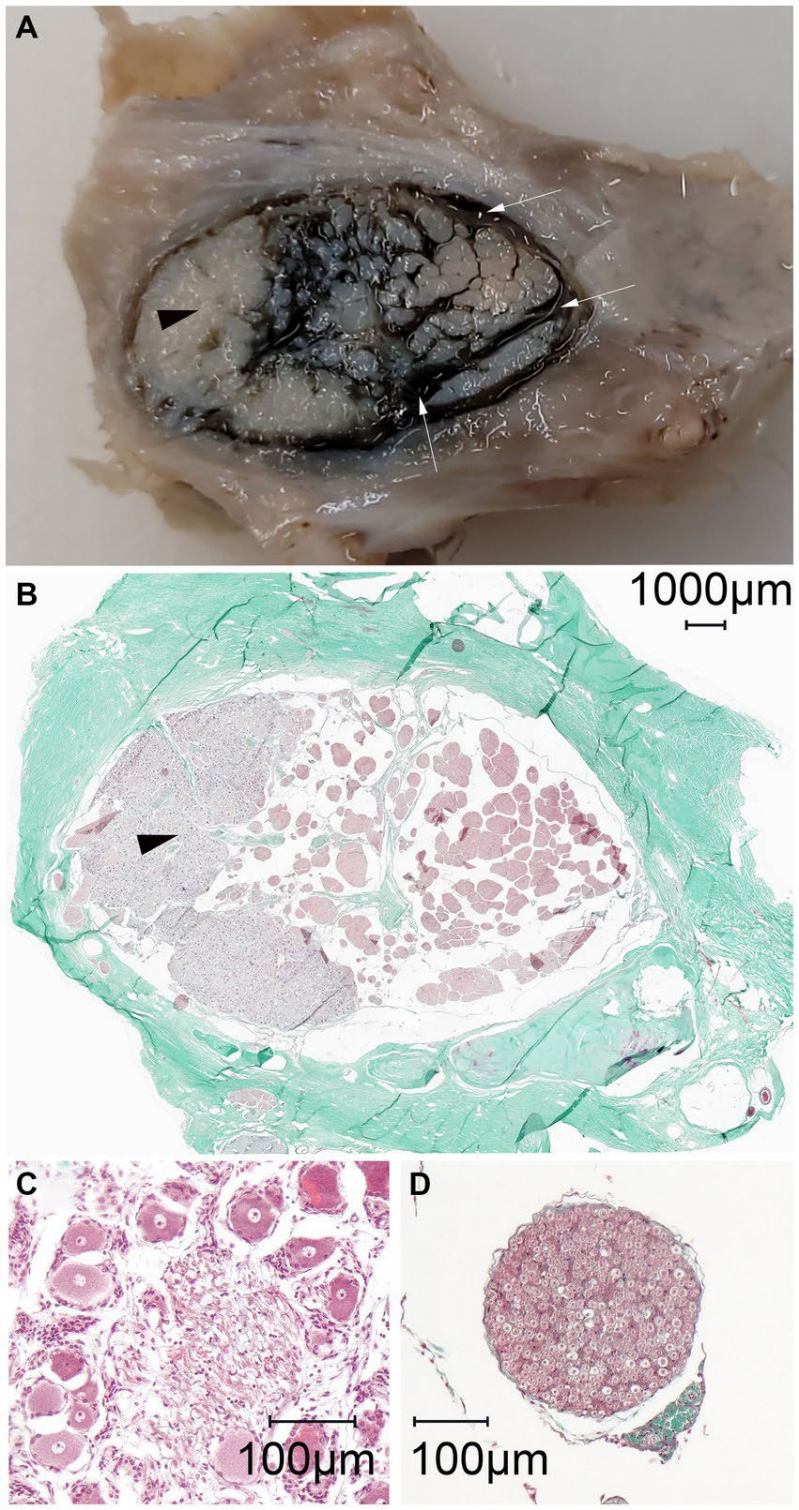
Macroscopic and microscopic representation (magnification: 100×) of the identical left equine cadaveric trigeminal cave. **(A)** The trigeminal cave was sliced parallel to the porus trigeminus, caudal view, medial is to the right. The lateral aspect of the trigeminal cave contains the densely packed trigeminal ganglion (black arrowhead). In the medial aspect, the fibers of the trigeminal plexus are surrounded by the dark green stained casting material (μAngiofil, white arrows). **(B)** Histologic slide of **(A)**. The casting material has been washed out during processing of the histology slides. The subarachnoid space is visible as a blank space surrounding the fibers of the trigeminal plexus. **(C)** Close-up of the ganglion showing the large cell bodies with prominent nuclei. **(D)** In comparison, a nerve fascicle surrounded by perineurium.

**Figure 7:**
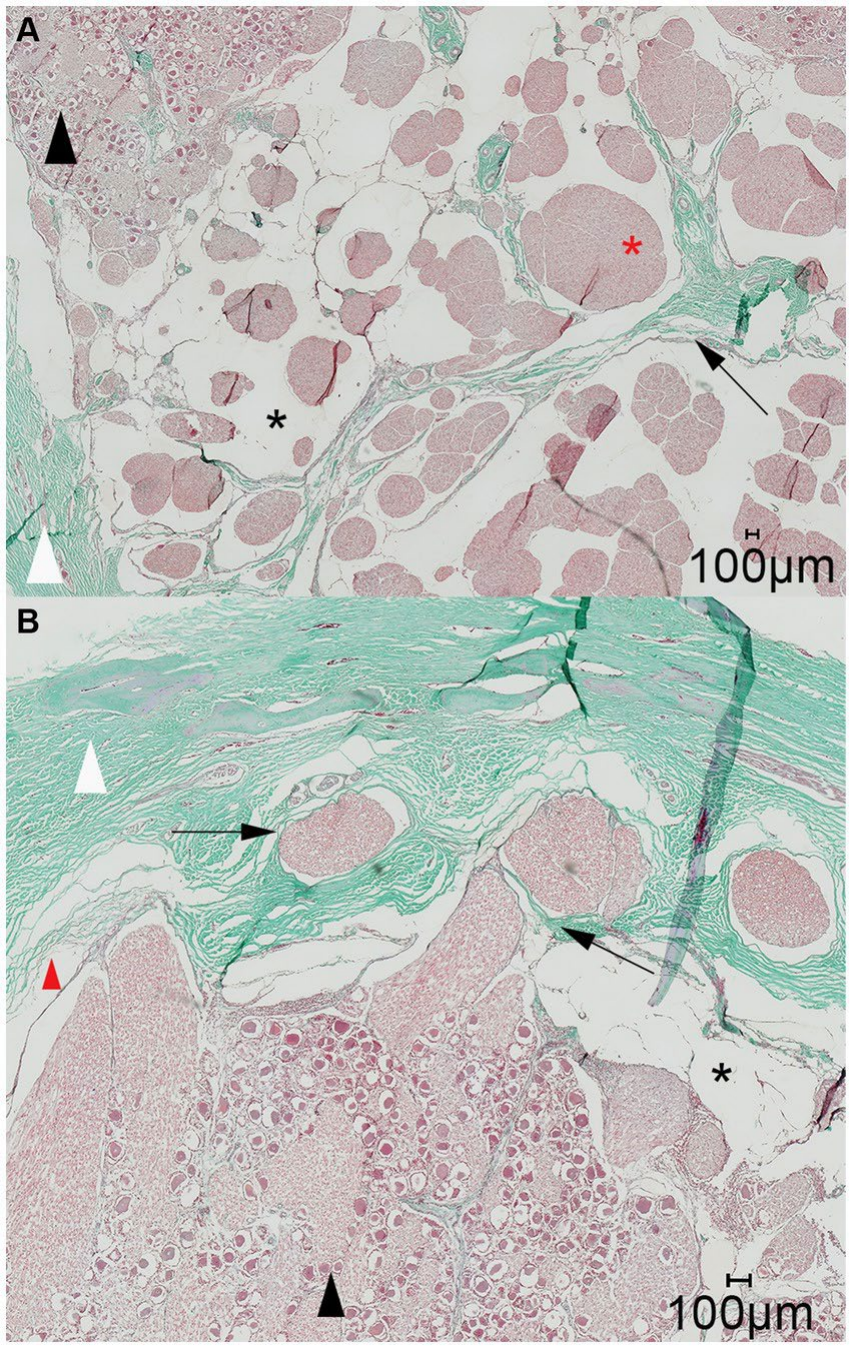
Representative light microscopic image of the trigeminal cave; Masson-Goldner trichrome staining; magnification 200×. **(A)** A dural septum (black arrow) from the dura mater (white arrowhead) and the closely adhered arachnoid membrane project into the trigeminal cave and between the fibers of the trigeminal nerve (red asterisk). The subarachnoid space (black asterisk) is visible as a wide blank space between the nerve fibers and the arachnoid trabeculations. **(B)** Nerve fiber bundles of the peripheral nerves (black arrows) emerging from the trigeminal ganglion (black arrowhead) separately perforate the dura mater through the cribriform area. During preparation of the histological slides, the arachnoid detached from the dura mater (red arrowhead). This is an artifact and should not be confused with the subarachnoid space (black asterisk).

The distribution of the casting material within the subarachnoid space was histologically assessed in three specimens. This included two specimens with a normal distribution pattern and one specimen with poor distribution, due to the suspected inadvertent perforation of the meninges. On all histologic slides shrinkage artifacts occurred causing detachment of the arachnoid from the dura mater. Gross inspection of the transverse and sagittal cross-sections of the formalin-fixed blocks prior to slicing for histology revealed a trabecular accumulation of the colored polymer-based casting material (μAngiofil) surrounding gray nerve bundles (Figure 6). The solid μAngiofil was subsequently washed-out upon preparation of the slides for histologic examination, thus precluding its microscopic detection. However, the subarachnoid space where the polymer had accumulated was still clearly visible as a blank space, forming a trigeminal cistern surrounding the nerve fascicles and enclosed by the arachnoid membrane. The pattern of the trigeminal cistern matched with the trabecular structure of the nerve fascicles surrounded by the green polymer visible on the formalin-fixed block, confirming the injection of the casting material into the subarachnoid space (Figure 6). On slides of both cutting orientations (parallel and perpendicular to the porus trigeminus), dural septa projected into the trigeminal cistern followed by arachnoid trabeculations enveloping the trigeminal nerve fibers (Figure 7).

**Figure 8:**
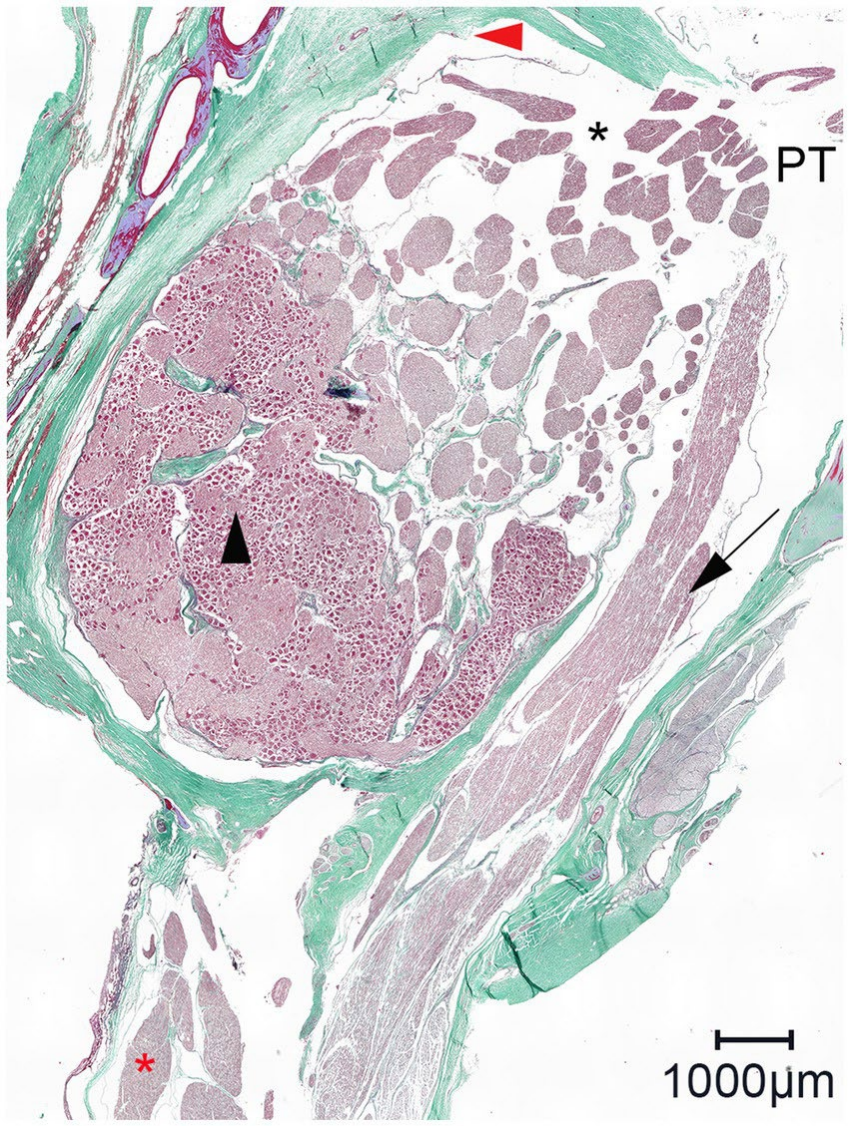
Representative light microscopic image of the trigeminal cave cut perpendicularly to the porus trigeminus; Masson-Goldner trichrome staining; magnification 100×. In the trigeminal cave, the motor root of the mandibular nerve (black arrow) runs in its own dural sheath, i.e., it is separated from the trigeminal ganglion by a dural septum, which becomes thinner toward the porus trigeminus (PT). Black arrowhead: trigeminal ganglion; red arrowhead: artifact due to detachment of the arachnoid from the dura mater; black asterisk: subarachnoid space; red asterisk: nerve fascicles of the mandibular nerve.

On the histologic slides of both cutting orientation, the nerve fascicles separately perforated the dura mater at the lateral aspect of the trigeminal cave (Figure 7). After running through the porous membrane, the nerve fascicles united to form the peripheral nerve. On histologic slides oriented perpendicularly to the porus trigeminus, additional nerve fascicles traveling within the trigeminal cave from the porus trigeminus at the inferior aspect of the trigeminal cave in a lateroventral direction ventral to the trigeminal ganglion toward the mandibular nerve were observed. At the level of the trigeminal ganglion these nerve fascicles appear to be separated from the trigeminal ganglion by a dural septum (Figure 8).

## 4 Discussion

The results of this cadaveric study confirm the presence of a subarachnoid space forming a cistern in the trigeminal cave of the horse. On MR images, a fluid accumulation of similar intensity to CSF closely surrounds the trigeminal ganglion, suggesting the presence of a subarachnoid space. In this study, this space was successfully injected with a radiopaque casting material, as confirmed by the similar distribution patterns of the CSF on MR images and of the casting material on CBCT-contrast cisternography and verified by subsequent histological examination of the trigeminal ganglion. Microtomographic examination of the casted subarachnoid space provided additional information about its extent, and about the diffusion pattern of the casting material within the trigeminal cave and along the main branches of the trigeminal nerve.

In humans, the segments of the trigeminal nerve contained within the trigeminal cave are the trigeminal ganglion and the trigeminal plexus (Figure 1) (7). The subarachnoid space within the trigeminal cave is located posteriorly to the trigeminal ganglion as the arachnoid is thought to tightly adhere to the trigeminal ganglion at its anterior surface (Figure 1) (6). However, a consensus on the meningeal architecture has not yet been reached and three models of the relationship between the meninges and the trigeminal ganglion have been discussed to describe the course and end of the arachnoid membrane and the dura mater around the trigeminal ganglion and its divisions (18, 19). Based on the distribution of the contrast agent in the subarachnoid space as determined by microCT analysis, it was possible in this study to divide the equine trigeminal nerve into two parts, as observed in humans (7). The trigeminal ganglion was located rostrally within the trigeminal cave. Upon contrast cisternography and microCT this was characterized by a lack of contrast agent due to its high tissue density, obviously not allowing penetration of the contrast agent. The trigeminal plexus was located within the caudal two thirds of the trigeminal cave near the porus trigeminus and viewed as increased accumulation of contrast agent with longitudinal filling defects and a trabecular pattern. The latter indicated that the trigeminal plexus is located within a widening of the subarachnoid space, comparable to the human trigeminal cistern. In the cistern in horses, the subarachnoid space containing the CSF was not only situated at the periphery of the nerve, but also between the nerve fascicles, i.e., the trigeminal plexus (Figure 1). On MR images, it was equally observed that the fluid arranged in a trabecular pattern between nerve fascicles and the trigeminal ganglion. This explained the trabecular pattern of the distribution of the contrast agent in this region. When the nerve fascicles converged rostrally to form the trigeminal ganglion, the contrast agent column was displaced toward the axial periphery of the trigeminal ganglion, and even disappeared focally (mainly abaxially) due to the adherence of the arachnoid membrane to the ganglionic tissue.

In humans, the volume of the trigeminal cave can be assessed radiologically on preoperative MR images and amounts to a mean volume of 0.4 mL. However, this volume refers to the whole volume of the trigeminal cave including the trigeminal nerve and the ganglion (20). In patients undergoing a glycerol rhizotomy, the estimated volume of glycerol to be injected can be intraoperatively assessed by performing a contrast cisternography under fluoroscopic guidance, i.e., by filling the trigeminal cave with a radiopaque contrast agent until overflow of the trigeminal cistern into the cranial cavity is observed. There, the mean reported volume of the trigeminal cistern is 0.25 mL, and rarely exceeds 0.4 mL (13).

In the present study, the overall mean volume of the equine subarachnoid space within the trigeminal cave was 0.34 mL. In experimental and clinical studies reporting on glycerol rhizotomy procedures in horses, a volume of 1.6–1.9 mL of glycerol was injected (14, 15). According to the results of the present study, this injected volume probably exceeds the capacity of the trigeminal cave in the horse. Interestingly, the volume of the trigeminal cave in horses and in humans is comparable. The area innervated by the trigeminal nerve is wider in horses, thus the number of nerve fascicles is larger, resulting in a larger cross-section of the trigeminal ganglion. Indeed, Newton counted about 500 nerve fascicles and a mean of approximatively 1.5 million fibers in the normal equine trigeminal root (4), whereas about 50 nerve fascicles and 150,000 fibers were counted in the trigeminal root in humans (21, 22).

The anatomy of the trigeminal cave has already been studied in other animal species. Kanpolat established a percutaneous approach to the trigeminal ganglion in dogs comparable to the surgical technique in humans (23) based on which the trigeminal ganglion was examined histopathologically after injection of 0.15 mL of glycerol into the trigeminal ganglion (24). Interestingly, Isik et al. reported that a subarachnoid space around the trigeminal ganglion does not exist in dogs (24). In contrary to this, Santifort et al. described leptomeninges and a subarachnoid space around the trigeminal nerve roots and the trigeminal ganglion in dogs comparable to the trigeminal cave in humans (25). Even though the trigeminal cave in dogs was subtle, they also suspected individual variability in morphology of the canine trigeminal cave (25). However, in these studies, no volumetric measurements of the trigeminal cave have been undertaken (23–25). Lunsford et al. injected glycerol into the trigeminal cistern of cats to investigate the electrophysiological and histopathological effects of glycerol. Based on the macroscopic assessment of the size and location of the trigeminal ganglion and nerve, they selected a volume of 0.05 mL of glycerol but did not assess the morphology of the trigeminal ganglion and a trigeminal cave on a microscopic level (26). More recently, Herta et al. conducted an anatomical study on rabbits to provide guidelines for percutaneous operations on the trigeminal ganglion in this species as an animal model for human research (27). They described the presence of a closed trigeminal cistern that was injected with 0.5 mL of glycerol. However, they observed an outflow of contrast agent into the posterior fossa and out of the injecting cannula when performing the contrast cisternography with this volume, suggesting that smaller amounts of glycerol should be injected in rabbits (27).

On microCT scans of the trigeminal cave in our study, the contrast agent was distributed in 7/12 specimens along the proximal aspects of the peripheral nerves, mainly along the mandibular nerve. Accordingly, a continuity between the contrast agent in the trigeminal cistern and around the peripheral nerves could be observed. On MR images, CSF accumulation around the peripheral nerves was not observed. Therefore, it could be argued that distribution of the contrast agent along the peripheral nerves was caused by excessive pressure applied while injecting the polymer, possibly creating an artificial enlargement of the subarachnoid space around the trigeminal divisions. However, the porus trigeminus represents the path of least resistance for the polymer, which explains the backflow occurring during the injection process and decreases the likelihood of excessive pressure in the trigeminal cave during the injection.

The most likely explanation for the contrast agent distribution around the peripheral nerves lies in the specific meningeal architecture of the trigeminal cave of horses. In humans, the peripheral sheaths of the three trigeminal divisions are a direct continuation of the meningeal wall of the trigeminal cave (28, 29). Extracranially, the dural sheaths of the three divisions fuse with the epineurium (28). The trigeminal cave and the dural sheath of the peripheral nerves are separated by a cribriform area, a porous membrane through which the nerve rootlets pass (28, 29). Interestingly, the motor root of the mandibular division enters a separate sheath in the inferior wall of the trigeminal cave whereafter it converges with the peripheral dural sheath of the mandibular nerve (28). In horses, histological evaluations of the trigeminal ganglion have been performed in several studies. Yet, the microanatomic meningeal architecture of the trigeminal cave and of its branching nerves has still not been thoroughly investigated (4, 14, 30, 31). The histologic findings in this study support the presence of a cribriform area, which could explain the distribution of the contrast agent around the peripheral nerve. The motor root of the trigeminal nerve also appeared to emerge from the trigeminal cave into the periphery in a separate dural sheath in horses (Figure 8). However, the histological sample size was too small for an accurate description of the course of the motor root and therefore warrants further investigation.

The distribution of contrast agent around the peripheral nerve rootlets can be clinically relevant when attempting a glycerol rhizotomy. To the authors’ knowledge, a peripheral distribution of glycerol following injection into the trigeminal cave has not yet been reported in humans. Little is also known about the mode of action and degradation of glycerol, but outflow of glycerol is likely to occur the same way as CSF. Based on the results of the present study, an accumulation of glycerol around the peripheral nerves can be expected, potentially leading to a longer duration of action on the nerve cells. In the case of the purely sensory nerves of the ophthalmic and maxillary divisions, this may well be desirable, as it could possibly lead to a higher cytotoxic effect and thus a better treatment outcome. Nevertheless, the effect of a prolonged contact between the glycerol and the mandibular nerve, which contains the motor root (Figure 1) responsible for the masticatory function (i.e., innervating the masseter, temporal and pterygoid muscles) remains to be elucidated. When glycerol is injected into the trigeminal cistern, contact between glycerol and the mandibular nerve and its motor root could still occur. In humans, masticatory dysfunction occurred as a complication after radiofrequency trigeminal rhizotomy in 4.1% of patients in one study (32), and after balloon compression (in 7% of the patients) but not after glycerol rhizotomy (33, 34). Winter et al. did not observe masticatory dysfunction in the eight horses undergoing percutaneous glycerol injection into the trigeminal ganglion (14). However, in this study, the accuracy of injecting needle placement was controlled on CT and not on MR images. Therefore, it could not be ascertained if the tip of the needle was positioned in the trigeminal cistern, and that glycerol accumulation within the trigeminal cistern and possibly distribution around the mandibular nerve occurred. Consequently, further research is required to determine whether glycerol would induce masticatory dysfunction when injected into the trigeminal cistern in a clinical setting.

Nonetheless, the results presented in this study show that a subarachnoid space forming a cistern in the trigeminal cave exists and that it could be targeted for a glycerol rhizotomy procedure in the horse. The trigeminal cistern is located dorsal to the lateral edge of the foramen lacerum, directly rostral to the internal acoustic meatus. This means that the largest fluid accumulation within the trigeminal cistern is located on the axial aspect of the trigeminal cave and remote to dense ganglionic tissue. Moreover, this part of the trigeminal cistern could be reached through the foramen lacerum via a direct percutaneous approach.

One limitation of the present study is inherent to its experimental cadaveric nature. The contrast cisternography was performed on fresh cadaver heads, disarticulated at the atlanto-occipital joint, and after removal of the calvaria. The resulting loss of the physiologic intracranial pressure, combined with the collapse of the subarachnoid space due to the outflow of CSF, could have caused the inadvertent penetration of the arachnoid during injection of the contrast agent in two specimens, in which the contrast agent accumulation within the trigeminal cave was ill-defined and distributed into the surrounding soft tissues structures. To minimize the risk of inadvertent penetration, the tip of the 25G hypodermic needle was blunted and slightly bent to follow the contour of the trigeminal ganglion. In live horses, a physiologic intracranial pressure and a non-collapsed, CSF-filled subarachnoid space could result in a greater CSF volume within the trigeminal cave, thus facilitating the injection and avoiding early outflow of glycerol into the posterior fossa.

To guarantee complete replacement of the CSF by the polymer-based contrast agent during injection, the cadaver heads were kept in a tilted position, ensuring that the trigeminal cave was as vertically oriented as possible while the porus trigeminus remained horizontal. As the injected contrast agent has a higher density than CSF and is hydrophobic, it progressively accumulates in the trigeminal cave and expulses the CSF out of the trigeminal cave into the posterior fossa. Furthermore, the vertical orientation of the trigeminal cave during injection prevented inadvertent outflow of the contrast agent during solidification of the polymer. However, the head orientation was only based on empirical observations during preliminary cadaveric trials, and the effect of other head orientations on the distribution pattern and on the volumetric assessment of the contrast agent was not assessed. Moreover, the inclusion of cadaver heads from different breeds, with slightly variable conformations, precluded the exact same positioning during every injection. This could have resulted in early outflow of the contrast agent from the trigeminal cave and residues of CSF within the trigeminal cave, thus inducing an underestimation of the microtomographic volumetric measurement of the trigeminal cave. This underestimation could have been potentiated by the entrapment of air bubbles within the contrast agent, which were occasionally detected within the trigeminal cistern on microtomographic slices.

Further limitations of this study include the small sample size and the heterogeneity of the cadaveric specimens. Although all horses included were adult and of comparable size and weight, breed-specific anatomical variations regarding the shape and the size of the trigeminal cave cannot be ruled out. Furthermore, no information can be provided about the volume of the trigeminal cave in ponies, draft horse breeds or other equids. Additional studies are needed to assess differences among breeds and to investigate if a correlation between horse size and the volume of the trigeminal cave exists. The first cadaver head was stored frozen after the MRI study. However, as the freezing and thawing process could impair the histologic assessment of the meninges, subsequent heads underwent all above-described procedures immediately after MR imaging. No differences in size, volume or contrast distribution were observed in the frozen head compared to the subsequent cadaver heads.

In conclusion, despite several decades of research on trigeminal-mediated headshaking, there is still no safe and reliably effective treatment for equids suffering from this painful condition. The development of novel therapy options is crucial to help the most severely affected horses, especially those that are refractory to medical management. This anatomical study is the first detailed description of the equine trigeminal cave and its boundaries, and thus an important basis for the development of a controlled and reliable rhizotomy technique in horses. Further studies are needed to assess the suitability of the equine trigeminal cave as a primary target structure for glycerol rhizotomy.

## Data availability statement

The original contributions presented in the study are included in the article/Supplementary material, further inquiries can be directed to the corresponding author.

## Ethics statement

Ethical approval was not required for the studies involving animals in accordance with the local legislation and institutional requirements because this research was performed on cadaver parts of animals slaughtered or euthanized for reasons unrelated to this study and therefore exempt from ethical review according to the guidelines from the Kanton of Bern, Switzerland. Written informed consent was obtained from the owners for the participation of their animals in this study.

## Author contributions

RB: Conceptualization, Investigation, Methodology, Visualization, Writing – original draft. KH: Conceptualization, Methodology, Supervision, Writing – review & editing. CP: Conceptualization, Methodology, Writing – review & editing. O-ZK: Methodology, Writing – review & editing. RH: Methodology, Writing – review & editing. CK: Conceptualization, Writing – review & editing. SK: Conceptualization, Supervision, Writing – review & editing. MP: Conceptualization, Investigation, Methodology, Supervision, Writing – review & editing.
