# Correction: An anatomical study of the subarachnoid space surrounding the trigeminal ganglion in horses—in preparation for a controlled glycerol rhizotomy in equids

**DOI:** 10.3389/fvets.2025.1749889

**Published:** 2025-12-15

**Authors:** Richard Becker, Kati Haenssgen, Christina Precht, Oleksiy-Zakhar Khoma, Ruslan Hlushchuk, Christoph Koch, Sabine Kaessmeyer, Mathieu de Preux

**Affiliations:** 1 Department of Clinical Veterinary Medicine, Vetsuisse-Faculty, Swiss Institute of Equine Medicine (ISME), University of Bern, Bern, Switzerland; 2 Division of Veterinary Anatomy, Department of Clinical Research and Veterinary Public Health, Vetsuisse-Faculty, University of Bern, Bern, Switzerland; 3 Division of Clinical Radiology, Vetsuisse-Faculty, University of Bern, Bern, Switzerland; 4 Institute of Anatomy, University of Bern, Bern, Switzerland; 5 Graduate School for Health Sciences, University of Bern, Bern, Switzerland

**Keywords:** trigeminal-mediated headshaking, trigeminal cave, Meckel's cave, magnetic resonance tomography, contrast cisternography, microtomography, histology, rhizotomy

Affiliation Graduate School for Health Sciences, University of Bern, Bern, Switzerland was omitted for author Mathieu de Preux. This affiliation has now been added for author Mathieu de Preux.

The original version of this article has been updated.

