# Supplementary material for: An anatomical study of the subarachnoid space surrounding the trigeminal ganglion in horses—in preparation for a controlled glycerol rhizotomy in equids
Source: Front Vet Sci. 2024 Jul 18;11:1424890. doi: 10.3389/fvets.2024.1424890 (PMC11291345; doi:10.3389/fvets.2024.1424890)
Supplement: Supplementary file 5 [file Table_1.pdf]

frontiers

Supplementary Material

An anatomical study of the subarachnoid space surrounding the trigeminal ganglion in horses - in preparation for a controlled glycerol rhizotomy in equids

Richard Becker[1], Kati Haenssgen[2], Christina Precht[3], Oleksiy-Zakhar Khoma[4], Ruslan Hlushchuk[4], Christoph Koch[1], Sabine Kaessmeyer[2], Mathieu de Preux[1]

*** Correspondence:**

Richard Becker, Swiss Institute of Equine Medicine, Länggassstrasse 124, Postfach 8466, CH-3001 Bern, Switzerland. Email: richard.becker@rubecker.de

Mathieu de Preux, Swiss Institute of Equine Medicine, Länggassstrasse 124, Postfach 8466, CH-3001 Bern, Switzerland. Email: mathieu.depreux@unibe.ch

1 Supplementary Videos

Supplementary Video 1. Microcomputer tomographic scan of the subarachnoid space of a right sided trigeminal cave. In the transverse section plane, the contrast accumulation is mostly present on the axial aspect of the subarachnoid space.

Supplementary Video 2. Microcomputer tomographic scan of the subarachnoid space of a right sided trigeminal cave, medial view. Sagittal section through the subarachnoid space showing the trabecular filling defects within the casting material.

Supplementary Video 3. Microcomputer tomographic scan of the subarachnoid space of a right sided trigeminal cave, dorsal view. Dorsal section through the subarachnoid space showing increased accumulation of casting agent on its axial aspect and the trabecular filling defects within the casting agent.

Supplementary Video 4. Microcomputer tomographic scan of the subarachnoid space of a left sided trigeminal cave. The contrast agent not only distributes around the origin of the mandibular nerve, but also extends along the course of the mandibular nerve through the foramen lacerum and along the nerves branching off outside the cranial cavity.
